# The economic burden of adult asthma in Cyprus; a prevalence-based cost of illness study

**DOI:** 10.1186/s12889-017-4184-0

**Published:** 2017-03-16

**Authors:** Savvas Zannetos, Theodora Zachariadou, Andreas Zachariades, Andreas Georgiou, Michael A. Talias

**Affiliations:** 1 DG European Programmes, Coordination and Development, 29 Byron Avenue, 1096 Nicosia, Cyprus; 2 Open University of Cyprus, 33 Giannou Kranidioti Avenue, 2220 Latsia, Nicosia, Cyprus; 3 Engomi Primary Heath Care Centre, Nikou Kranidioti & Macedonia, Nicosia, 2411 Cyprus; 4 American Medical Centre, 215, Spyrou Kyprianou Avenue, 2047 Nicosia, Cyprus; 5 Respiratory Department Clinic, Nicosia General Hospital, 215 Nicosia – Limassol Old Road, 2029 Strovolos, Nicosia, Cyprus

**Keywords:** Cost of illness, Asthma, Burden of disease, Cyprus

## Abstract

**Background:**

Asthma is one of the main non-infectious diseases of the respiratory system with substantial economic burden worldwide. The objective of this study was to estimate the economic burden of adult asthma in Cyprus during 2015.

**Methods:**

A retrospective probabilistic prevalence-based cost of illness model was developed to calculate the economic burden of asthma including direct and indirect costs. The bottom-up approach (person-based data) was used for the calculation of direct costs while for the calculation of indirect costs the approach of human capital was employed. In addition, bootstrapped sensitivity analysis with 1000 bootstrap simulations was performed in order to calculate a 95% Confidence Interval (CI).

**Results:**

Mean patient cost of asthma in Cyprus in 2015 was estimated at €579.64 (95% CI: €376.90–€813.68). Direct costs accounted for 82.08% of the overall expenses, €475.75 per patient (95% CI: €296.94–€697.69). Indirect costs of €103.89 (95% CI: €49.59–€181.46) accounted for 17.92% of the overall expenses.

**Conclusion:**

This was the first study in Cyprus, which used bootstrapped prevalence-based cost of illness model to estimate the cost of asthma. This study confirms that asthma is an expensive disease for the society. In addition, it provides important information and analysis of the economic consequences of asthma to policy makers in order to strengthen surveillance of the disease as well as draft the national health policy accordingly.

**Electronic supplementary material:**

The online version of this article (doi:10.1186/s12889-017-4184-0) contains supplementary material, which is available to authorized users.

## Background

Asthma is one of the main non-infectious diseases of the respiratory system. It is a chronic, inflammatory disease of the airways characterized by variable expiratory airflow limitation and recurrent episodes of breathlessness, wheezing, cough and chest tightness [1]. According to the World Health Organization, 235 million persons are currently suffering from asthma worldwide. Furthermore, it is a common disease that affects all countries, low income and high income countries. Approximately, 80% of all deaths due to asthma occur in low and middle income countries. Asthma is often under-diagnosed and under-treated thus creating significant burden to the individuals and their families as well as to the society as a whole [2]. World Health Organization estimates mortality of asthma at around 250 000 deaths per year [3]. High mortality occurs in countries where access to drugs is relative low [3].

Asthma is one of the most costly chronic diseases, both in the developed and developing world [4]. It is a major factor for the use of healthcare services, particularly the emergency services and the prescription drugs. In the future, the cost is expected to increase significantly [4]. As a result, the Global Asthma Report (2014) recommends that all Governments should estimate the economic cost of asthma in their countries, including healthcare costs and productivity losses [5]. Overall, on average, asthma accounts for 1–2% of total healthcare costs in developed countries [6].

Although no official data exist for the prevalence of asthma in Cyprus, the National Statistical Service, in 2008, estimated the prevalence of asthma at around 5.1% [7]. This is a self-reported percentage for the year 2008. Given that many different countries had reported a rapid increase in the prevalence of asthma [8–10] and the fact that it is generally accepted that asthma is a mis-diagnosed disease [2], the prevalence of asthma in Cyprus can be assumed to be higher. Thus, as recommended [5], it is extremely important for the Cyprus Healthcare system to estimate the economic burden of the disease in order to understand its economic impact and to draft its national policy accordingly.

Cost of illness studies have as an objective to evaluate the burden on the society due to the disease. These studies are descriptive, they provide economic values and summarise the costs of a particular disease. Their goal is to present the economic burden of a disease. Therefore, researchers should, recognise, record and measure the value of costs of the disease [11]. Disease costing studies present useful opportunities for communication with both the public and policy makers about the importance of particular diseases [12] so as to draft their policies accordingly.

## Methods

### Objective

The objective of this study was to identify and estimate the economic burden of adulthood asthma in Cyprus during 2015, which consists of the direct medical costs as well as the indirect costs relating to the damage caused to society due to absenteeism from work.

### Study design

This was a retrospective approach costing study, based on prevalence, focusing on both direct and indirect costs of the disease. Person-based data (bottom-up approach) was used for the calculation of direct costs. In addition, indirect costs were calculated using the human capital approach. The study uses the societal perspective which covers all aspects of costs such as direct medical, mortality, and indirect economic costs.

Studies based on prevalence estimate the treatment due to illness in a given year and the costs resulting from this treatment. According to Tarricone et al. [13], cost of illness studies based on prevalence are particularly useful when the main purpose of the study is to warn the policy-makers the economic burden of a disease has been somewhat underestimated. In addition, such studies will guide them to design cost containment policies due to the fact that these studies provide managers a picture of the overall burden and more importantly, the major cost components, i.e. areas where cost containment policies will have the greatest impact [13].

The human capital approach was implemented in order to evaluate the indirect economic burden of asthma in Cyprus. The human capital approach assumes the perspective of the patient and takes into consideration every man-hour that was not worked by the patients as a corresponding loss in productivity [14]. Indirect costs of each patient depend on income and the overall number of sick leaves from work resulting from nursing and taking care of the patient. Income per capita, as declared by the Ministry of Finance, was used to calculate the lost income [15].

### Time frame and data

The study population consisted of all adults living in Cyprus in 2015. The methodology included two stages. In stage I, subjects were contacted by telephone and were asked a screening questionnaire that included questions about symptoms suggestive of asthma, the use of any medication for asthma, as well as symptoms that suggest the presence of hay fever and nasal allergies. In stage II, a random sample of subjects who undergone the screening questionnaire and indicated symptoms of asthma were asked to participate to a more detailed interviewer-led questionnaire, skin-prick test (SPT), blood tests for the measurement of total and specific immunoglobulin-E (IgE) and spirometry.

### Questionnaires

The screening questionnaire was developed from the European Community Respiratory Health Survey (ECRHS) [16]. It was translated in Greek and validated using the appropriate methodology [17, 18] (Additional file 1). The interviewer-led questionnaire was based on ECHRS II main questionnaire and followed the recommendations of Asthma Outcome Workshop [19] (Additional file 2). The questionnaire included, among others questions relating to asthma symptoms, questions about emergency department visits, hospital stays, outpatient visits, asthma medications, asthma-related ancillary services and finally absenteeism from work due to asthma.

### Sample

For Stage I (Screening Questionnaire), subjects were a representative sample of 18+ years old. Stratified random sampling was performed and gender, age and district of residence were defined as strata. Subjects were conducted by telephone and were asked if they wanted to answer the screening questionnaire. Overall, 1913 subjects answered the questionnaire (out of 8986 that were conducted giving a response rate of 21.29%) yielding a margin of error of ± 2.24%, at a Confidence Level of 95%.

Stage II (Main Questionnaire and further tests) had as an objective to provide a random sample of subjects to be studied. The random sample was selected from individuals who participated in Stage I and answered at least one “yes” to four pre-defined questions in the screening questionnaire. These questions were: “Has a doctor ever told you that you had asthma?” “Have you had an attack of asthma in the last 12 months?”, “Have you been woken by an attack of shortness of breath at any time the last 12 months?”, “Are you currently taking any medicine (including inhalers aerosols or tablets) for asthma?” According to ECHRS these questions exhibit high sensitivity in detecting asthma [16]. From the initial pool of 1913 subjects, 513 were randomly selected for the second stage of study. Of those, 200 (38.99% response rate) agreed to participate in stage II for the detailed evaluation at the Respiratory Clinic of each General Hospital throughout Cyprus. An interview-led questionnaire, spirometry (before and after bronchodilation) and IgE test as well as SPT were performed for all participants from pulmonologists.

### Diagnosis of the disease

Diagnosis was defined according to Expert panel report 3: guidelines for the diagnosis and management of asthma [20]. A workshop that included pulmonologists and a general practitioner examined all data (spirometry, blood tests, symptoms and personal and family history) for each subject and assessed wherever the disease was present or not. From the 200 subjects that were assessed at each Respiratory Clinic 36 were finally diagnosed with asthma. Relating to the fact that asthma is a disease that is often misdiagnosed [2] in our data 75% that self-reported as being asthmatic were not diagnosed with asthma by the doctor workshop based on the above mentioned examinations. Thus, it is extremely important to have medical diagnosis of asthma in order to estimate the burden of the disease accordingly.

### Cost components

#### Direct costs

Direct medical costs of asthma consist of hospital stays, outpatient visits, asthma medications, asthma-related ancillary services (inpatient and outpatient laboratory and radiology tests) and finally emergency department visits. As an example, the overall cost of hospitalisation of patients with asthma throughout 2015 was obtained by multiplying the total number of nights with the cost per night. For unit costs, market prices were used because of their reflection on the cost to society [21].

It is worth noting that only costs of diagnosis and treatment of asthma were included in the study and not any prevention costs. The reason for this is that prevention costs are dependent on each person’s decision. That is why, in general, prevention costs are viewed as discretionary and they are not normally included in the cost of illness studies [22, 23].

#### Indirect costs

From the point of view of society, any losses in productivity due to the disease have to be included in the cost of illness study. The human capital approach assumes that indirect costs represent the loss of production for the economy due to work absenteeism. Therefore, any losses in productivity due to the disease should be estimated. The Hanover Consensus states that these losses in productivity should be estimated without consideration of any differences in the occupation, gender or age, using the average gross income for the period studied [24]. Thus, indirect costs included production loss due to sick leaves, and hospitalizations. The cost of production loss was estimated from an average salary of €85 per day among Cypriot employees [25].

### Premature mortality

Premature mortality costs are derived by valuing potential years of life lost (PYLL) due to asthma before the usual retirement age, which in Cyprus is the age of 65. In order to have an estimate of average years of life lost per death, a division of the PYLL with the number of deaths should be performed. However, high degree of uncertainty affects premature mortality due to illness and it is considered biased valuation of lost life [23, 26]. Thus, costs relating premature mortality due to illness should be avoided or if calculated, they should be reported separately [23]. Furthermore, mortality costs include future losses and since this is a prevalence based cost of illness study the timeframe is inconsistent with the other costs [27]. Therefore, no productivity losses due to premature death are included in the calculations for the overall costs of asthma.

### Extrapolation of costs to society

This cost of illness study used bottom-up approach to quantify resource use. Bottom-up approach allocates the resources used and the productivity loss of individuals due to the disease. Thus, mean per-person costs can be extrapolated to the whole population bearing the disease by using the appropriate prevalence data [23]. The study includes a stratified random sample of patients that were diagnosed in 2015 with the disease and their characteristics are representative of the total population suffering from asthma. Thus, given that a stratified random sampling was applied in order to have a representative sample of the whole population and a bootstrapped sensitivity analysis that aimed to capture any other potential unaccounted effects, an extrapolation, based on the weights of each strata, of costs to society was performed.

### Sensitivity analysis

When risk and uncertainty exists, sensitivity analysis is always recommended [28]. Thus, cost of illness studies must always report and evaluate the results of such analysis [29]. It is of immense importance, a sensitivity analysis to be carried out that takes into consideration alternative values for all assumptions and important cost parameters in the study. Consequently, the aim of the sensitivity analysis is to construct a 95% Confidence Interval (CI) for all point estimates as recommended by literature [23]. Point estimates are particularly helpful to explain and to bring attention to the economic burden of an illness. However, an interval of possible costs has more reliability for health policy analysis [30, 31].

For the purpose of this study a deterministic (1-way) sensitivity analysis was performed on prevalence and a non-parametric stratified bootstrap analysis with 1000 simulations was performed on all cost components. The reported 95% CI can be considered as a measure of uncertainty of this estimate. In order to compute CI in the customary way, the distribution of the estimate must be known. However, to the best of our knowledge, this is the first study involving prevalence based costing of asthma in Cyprus, thus no prior information on distributions of costs is available or known. Therefore, the precision of the point estimate cannot be calculated using traditional statistical methods because the distribution of the cost estimate is analytically not known and our sample size was not large enough to estimate the distribution based on the sample. In this way there was no need for any proper statistical assumptions for the distributions of the data or for the cost estimates.

The fundamentals of bootstrapping were described by Enfron [32]. The procedure requires generating independent samples with replacement from the empirical distribution of the observed data. Equation 1 demonstrates the bootstrap procedure. Let ${\overline{\mathrm{x}}}^{*}$ the mean of each bootstrap sample and m the number of bootstrap samples. Then, the mean of 1000 bootstrap replications equals

1 $$ {\overline{x}}_{boot}=\frac{1}{m}{{\displaystyle \sum \overline{x}}}^{*}=\frac{1}{1000}{\displaystyle \sum {\overline{x}}^{*}} $$

The average (which is the estimate in question), is calculated for each of the replicate samples. Thus, there is an average for each replicated sample which yields a distribution for the estimate of the average. In our study, bootstrap estimates were obtained after a two-step calculation. In step 1, the distribution of the average is computed. In step 2, a point estimate and the respective 95% CI was calculated.

**Fig. 1:**
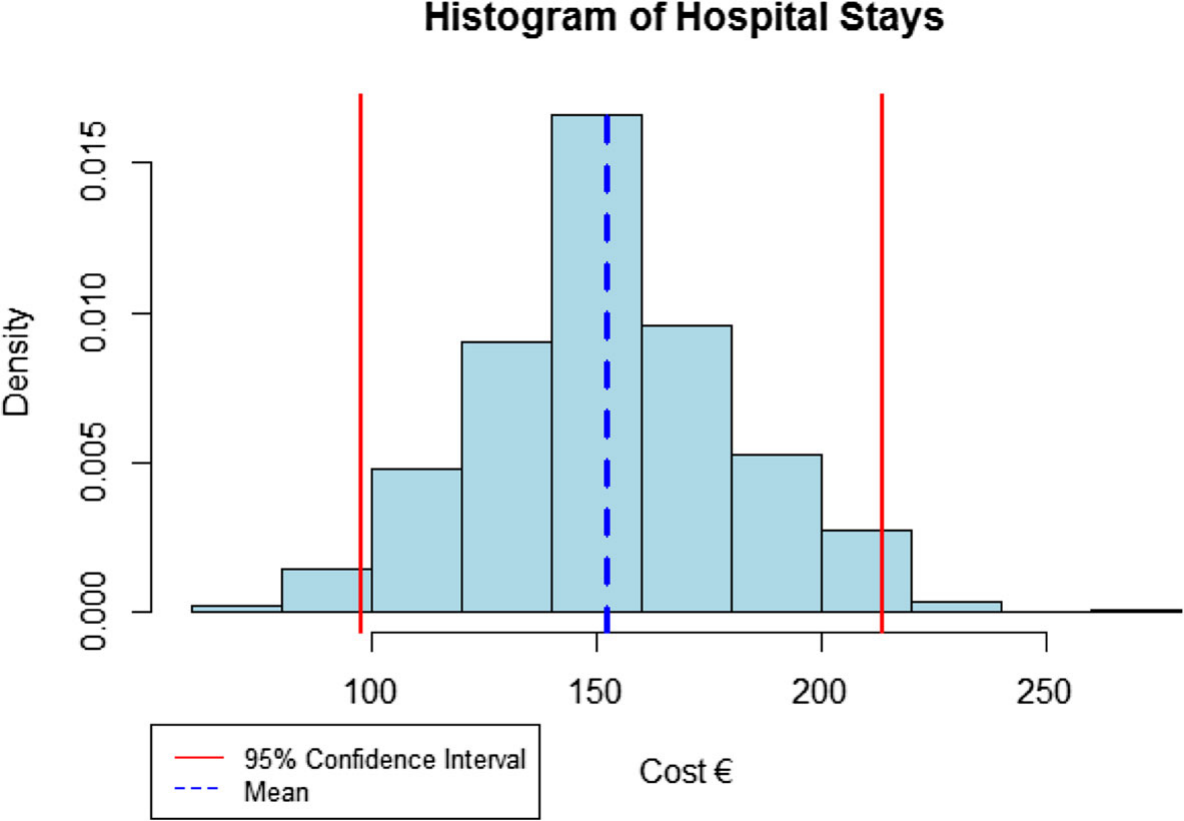
Histogram of the bootstrapped values of costs for hospitalisation per patient for 2015 with the corresponding 95% Confidence Interval

- Step 1
  In order to approximate the distribution of estimated average using a bootstrap sample, we calculated the averages for each replicated sample. Each sample was randomly selected and taken independently with replacement from the empirical distribution. According to the literature [33], 1000 replications are needed in order to have stable estimates. Therefore, 1000 samples were generated and a distribution of the weighted average of the cost in interest was estimated. Figure 1 illustrates the above by displaying the distribution of medication per patient. When the distribution is defined, all moments (i.e. variance, skewness, etc) can be estimated but we focused on the calculation of 95% CI as it is the measure of uncertainty as described above.
- Step 2
  Because of the large enough sample of replicates the mean of the estimated histogram is equivalent to the arithmetic mean of the sample. On the other hand, the lower and upper bounds of the 95% CI is directly derived from the histogram as an estimate of the 2.5 and 97.5% percentiles (P_2.5_ and P_97.5_) of the bootstrapped distribution of the cost in question, as shown in Fig. 1.

### Data analysis

In order to estimate costs Microsoft Excel 2007 [34] was used for the analysis. Bootstrap simulations for sensitivity analysis were performed in R v.3.2.2 [35].

## Results

Demographic characteristics of the sample diagnosed with asthma are presented in Table 1. The majority were males (61.1%) while almost four out of ten were university graduates (38.9%). Three out of four subjects used medication for asthma during 2015. In addition, around 40% is reported to have allergic rhinitis a percentage that was confirmed by blood tests (IgE and ECP). Mean age was 49.8 (±13.9) and Body Mass Index averaged at 28.3 (±4.6). Lastly, the reversibility of spirometry had a mean of 12.09% (±7.2%).

**Table 1:** Demographic characteristics of the study sample of patients with asthma (*N* = 36)

	Number	Percentage (%)
Gender		
Male	22	61.1
Female	14	38.9
Education		
High School	22	61.1
University	14	38.9
Area of Residence		
Urban	18	51.4
Rural	17	48.6
Do you have allergic rhinitis?		
Yes	14	38.9
No	22	61.1
IgE (IU/L)		
< 115	22	61.1
115+	14	38.9
ECP (ng/ml)		
< 20	22	61.1
20+	14	38.9
Have you used inhaled medication due to difficulty in breathing, wheezing, “whistling” chest or asthma crisis during the last year?		
Yes	27	75.0
No	9	25.0
	Mean	Standard Deviation
Age (years)	49.8	13.9
Body Mass Index (Kg/m^2^)	28.3	4.6
Reversibility %	12.10	7.2

### Cost estimates

Table 2 illustrates the mean costs per patient (direct and indirect) born by Cyprus State and the patients. More specifically, direct medical expenses were estimated at €475.75 and accounted for 82.08% of the overall expenses. Direct medical costs consist of visits to the doctor (€48.61 average per patient), ancillary services that included laboratory and radiology tests (€49.72 average per patient), medication (€207.97 average per patient), Emergency Department visits (€16.67 average per patient) and finally, hospitalization (€152.78 average per patient).

**Table 2:** Direct and indirect costs of patients with asthma during 2015 and a 95% Confidence Interval based on 1000 bootstrap samples

Type of Cost	Units	Unit Cost	Mean Cost per patient (€)	%	95% Confidence Interval (€)	
Direct Medical						
Visits to the doctor	35	€50.00/visit	48.61	8.39%	25.00	77.49
Ancillary Services^a^	n.a	n.a	49.72	8.58%	14.51	97.33
Medication^a^	n.a	n.a	207.97	35.88%	80.35	367.51
Emergency Department Visits	12	€50.00/visit	16.67	2.88%	2.44	33.33
Hospitalisations	25	€220.00/day	152.78	26.36%	98.63	214.76
Total			475.75	82.08%	296.94	697.69
Indirect						
Lost Income		€85/day	103.89	17.92%	49.59	181.46
Overall			579.64		376.90	813.68

*n.a* not applicable

^a^Includes all relevant laboratory and radiology tests/drugs of various prices

Further, the societal perspective accounts losses in productivity due to disease in cost estimates for a disease. As already mentioned, the human capital approach was used to estimate the indirect costs. An average of €103.89 of lost income per patient was estimated which consists of 17.92% of the total asthma cost.

The aggregated total cost of asthma to the society for 2015 is represented in Table 3. The overall cost of asthma to the society of Cyprus is estimated at €20,033,332. Direct medical expenses are estimated at €16,442,719. The most important expense is the medication cost estimated at €7,187,793. Finally, losses in productivity are estimated at €3,590,613, giving an overall cost of asthma to the society of €20,033,332.

**Table 3:** Annual total direct and indirect costs of asthma during 2015, in Cyprus

Type of Cost	Annual Cost (€)
Direct Medical	
Visits to the doctor	1,680,043
Ancillary Services	1,718,407
Medication	7,187,793
Emergency Department Visits	576,143
Hospitalisations	5,280,333
Total	16,442,719
Indirect	
Lost Income	3,590,613
Overall	20,033,332

### Sensitivity analysis

**Fig. 2:**
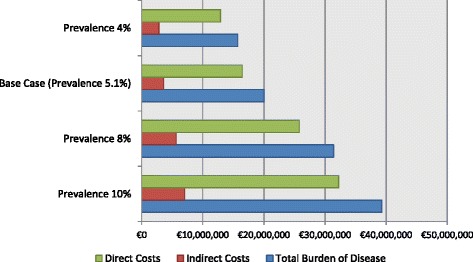
Sensitivity Analysis of total cost of asthma, 2015

Figure 2 demonstrates the results of one way (deterministic) sensitivity analysis. Prevalence rates were decided by the previous mentioned workshop of physicians taking into consideration the prevalence rates of neighbouring countries [36, 37]. Holding the above calculations constant, differences in prevalence increase the aggregate burden of the disease to the society at about €40 million. More particularly, at a prevalence rate of 10%, direct medical costs are estimated at €32,240,626, indirect costs at €7,040,418, thus giving an overall burden of the disease at €39,281,044.

Table 2 shows the 95% CI of the bootstrapped sensitivity analysis based on 1000 stratified bootstrap samples that was performed on each cost variable. The total direct medical expenses estimated at €475.75 per patient have a 95% CI of €296.94–€697.69 while the indirect cost amounted €103.89 (95% CI: €49.59–€181.46). The overall burden of the disease has a corresponding 95% CI of €376.90–€813.68.

## Discussion

This was the first study performed in Cyprus that evaluated the economic burden of asthma using the social perspective, with a bootstrapped prevalence-based approach. The study found the total cost of asthma per patient at €579.64 (95% CI: €376.90–€813.68). This cost includes medical direct costs (€475.75/patient, 95% CI: €296.94–€697.69) and indirect costs (€103.89/patient, 95% CI: €49.59–€181.46).

In the United States, the cost of asthma ranges from $3264 to $4912. Asthma is the 13^th^ most expensive medical condition and the 7^th^ leading cause of lost workdays in the United States [38]. More specifically, Colice et al [39] said that employers in the United States spend, on average, $1680 per year for people with asthma, for certain asthma related costs such as drug and medical costs. The overall increase in expenditure on health care per patient (direct cost) with persistent asthma was found to be $4412 while the indirect costs were $924 per person [39].

In a similar population, another study found that patients with asthma had three times higher medical claims than the average recipient of medical insurance and the total average annual per capita cost of the employer (including indirect costs) was about 2.5 times higher ($5385 against $2121) [40]. On the other hand, in a cross-sectional study in northern California the total per person annual cost of asthma was estimated at $4912 [41]. The bulk of the costs were the direct costs ($3180 or 65% of total) while the indirect costs were estimated at $1732 (35%).

Several European Union (EU) countries have also estimated the overall costs of asthma. More specifically, the total annual costs associated with asthma in Switzerland was estimated at around 1.2 billion Swiss francs per year [42]. Direct medical costs constituted 61% (762 million Swiss francs) and the remainder 39% for indirect costs. The majority of indirect costs (75%) represented the home care of patients with asthma. In Italy, the average annual cost per patient was €741 [43], with 43% of the total cost associated with the direct costs and 57% with the indirect costs [43]. In another Italian study among adult patients, the total cost of asthma was estimated €1260 [44]. These costs were allocated as follows: cost of drugs (16%), doctor visits (12%), emergency services and hospitalization costs (20%) and indirect costs (52%). The per patient annual total cost of asthma in Spain was estimated at $2879 [45]. Direct costs accounted for $885 (31%), of which prescription drugs accounted for 45%, ($400) and hospitalization accounted for 33%,($289). Indirect costs were estimated at $1993 mean per patient. In the Netherlands, the largest expense for asthma was prescription drugs [46].

Our study revealed that the highest cost for patients with asthma is the medication accounting for 35.88% of the overall cost of the disease and 43.70% of the direct costs. This is similar to the results in Italy where medication accounted for 47% of the direct costs [43], to the results in Spain where prescription drugs accounted for 45% of direct costs [45], the results in the USA where drugs accounted almost half of the direct costs [41] and to the Netherlands where medication was the largest expense [46]. Furthermore, this study revealed that indirect costs are significant and should not be overlooked. The indirect cost associated with asthma in Cyprus was estimated at €103.89 (95% CI: €49.59–€181.46) accounting for 17.92% of the overall costs. It is considerably low if compared with the abovementioned studies where indirect costs ranged between 35% in the USA [41] to 57% in Italy [43].

The cost of asthma has been estimated by several countries. The final cost of these estimates varies considerably, due to the differences in data collection, in their methodology, the monetary valuation of the included resources, the population included and the perspective of the cost of illness study. Furthermore, prices in healthcare vary between countries, thus, it is inappropriate to compare reported costs of each study. However, cost of illness studies expand our knowledge to the consequences (financial and economic) of asthma and provide essential information for further economic analyses of dealing with the disease and its medication [47].

This study, as with all cost of illness studies, has limitations. Cyprus has scarce availability of data. This is the reason why it is common to depend on sample data for health related research in Cyprus. However, every effort was employed to have a representative random sample and a bootstrapped sensitivity analysis was conducted so as to account for the differences in the selected sample. Another limitation of the study was that for the calculation of indirect costs, it only took into consideration work absenteeism. The productivity loss when employees do not optimally perform due to the illness when they are at work was not taken into consideration due to lack of information. Lastly, it should be noted that the calculations were based on a self reported prevalence rate, thus some uncertainty regarding asthma prevalence still exists in Cyprus.

### Recommendations

As noted above, our study revealed that the highest cost for patients with asthma is the medication accounting for 35.88% of the overall cost of the disease and 43.70% of the direct costs. A study of the pharmaceutical market in Cyprus revealed that Cyprus has as high pharmaceutical prices, especially when weighted with GDP [48]. Thus, an overall national policy of reducing the prices of drugs, especially the drugs used for chronic diseases is recommended as a policy measure to reduce asthma cost. Furthermore, training of physicians (especially General Practitioners, Internists as well as Pulmonologists) who are responsible for the diagnosis and management of asthma according to GINA guidelines is another important measure that would be beneficial for the patients and an indirect way of reducing costs. In addition, training patients towards self-management of asthma and medication adherence is another important measure that would lead to cost reduction. Finally, pharmaceutical services should apply a more flexible policy regarding the availability of newer asthma medication in the public sector pharmacies as well as access to physicians in prescribing those medications is of paramount importance for reducing the overall cost of asthma in Cyprus.

## Conclusion

This was the first study in Cyprus, which used bootstrapped prevalence-based cost of illness model to estimate the cost of asthma. This study confirms that asthma is an expensive disease for the society. In addition, it provides important information and analysis of the economic consequences of asthma to policy makers in order to strengthen surveillance of the disease as well as draft the national health policy accordingly.

## Additional files

Additional file 1: Screening questionnaire. (DOCX 22 kb)
Additional file 2: Interviewer-led questionnaire. (DOCX 26 kb)

## Abbreviations

CI: Confidence Interval
ECRHS: European Community Respiratory Health Survey
EU: European Union
IgE: Immunoglobulin-E
WHO: World Health Organisation
