# Supplementary material for: The economic burden of adult asthma in Cyprus; a prevalence-based cost of illness study
Source: BMC Public Health. 2017 Mar 16;17:262. doi: 10.1186/s12889-017-4184-0 (PMC5356320; doi:10.1186/s12889-017-4184-0)
Supplement: Additional file 1: — Screening questionnaire. (DOCX 22 kb) [file 12889_2017_4184_MOESM1_ESM.docx]

**Appendix 1**

1. Have you ever had wheezing or "whistle" in the chest in the last year?

YES __NO __

If no, go to question 2, if yes:

1.1. Did you have any difficulty in breathing when you had this "whistle” or wheezing in chest?

YES __NO __

1.2. Have you had this wheezing or whistling when you did NO YES not have a cold? YES __NO __

2. Have you woken up with a feeling of tightness in your chest at any time in the last year?

YES __NO __

3. Have you been woken by an attack of shortness of breath at any time the last year? YES __NO __

4. Have you ever told by a doctor that you have bronchial asthma or allergic rhinitis or allergic asthma?

YES __NO __

5. Have you had an asthma attack in the past year?

YES __NO __

6. Are you currently taking any medicine (including inhalers aerosols or tablets) for asthma ?

YES __NO __

7. Do you have an allergy nose like allergic rhinitis or allergic skin such as eczema, or allergic eye conditions such as allergic conjunctivitis?

YES __NO __

8. Do you smoke? YES __NO __

If no, go to question 9, if yes:

8) How many cigarettes a day? number ____

8b) For how many years? years ____

9. Smoking in the past? YES __NO __

If no, go to question 10 if yes:

9a) How many cigarettes a day? number ____

9b) For how many years? years ____

9c) When you stopped smoking? Year ________

10. Sex: Male __Female __

11. Age (years): ____
