# Supplementary material for: The economic burden of adult asthma in Cyprus; a prevalence-based cost of illness study
Source: BMC Public Health. 2017 Mar 16;17:262. doi: 10.1186/s12889-017-4184-0 (PMC5356320; doi:10.1186/s12889-017-4184-0)
Supplement: Additional file 2: — Interviewer-led questionnaire. (DOCX 26 kb) [file 12889_2017_4184_MOESM2_ESM.docx]

**Appendix 2**

1. Have you had wheezing or whistling in your chest at any time in the last 12 months? NO YES

IF 'NO' GO TO QUESTION 2, IF 'YES':

1.1 Have you been at all breathless when the wheezing noise was present? NO YES

1.2. Have you had this wheezing or whistling when you did not have a cold? NO YES

2. Have you woken up with a feeling of tightness in your chest at any time in the last 12 months? NO YES

3. Have you been woken by an attack of coughing at any time in the last 12 months? NO YES

4. Have you had an attack of shortness of breath that came on following strenuous activity at any time in the last 12 months?

5. Do you usually cough during the day, or at night, in the winter?

6. Have you ever had asthma?

IF 'NO' GO TO QUESTION 6.1, IF 'YES':

6.1 Was this confirmed by a doctor?

6.2 How old were you when you had your first attack of asthma?

6.3 How old were you when you had your most recent attack of asthma?

6.4 Have you had an attack of asthma in the last 12 months?

6.5 How many attacks of asthma have you had in the last 12 months?

7. Have you visited a doctor because of breathlessness or wheezing or "whistling" in the chest or difficulty in breathing or asthma attacks in the past year? NO YES

8. How many times did you see a doctor or other healthcare provider in the last 12 months?

9. Have you visited an Emergency Department because of breathlessness or wheezing or "whistling" in the chest or difficulty in breathing or asthma attacks in the past year?

10. How many times did you visit an Emergency Department in the last 12 months?

11. Have you stayed overnight in a hospital because of breathlessness or wheezing or "whistling" in the chest or difficulty in breathing or asthma attacks in the past year?

12. How many nights did you stay overnight in a hospital in the last 12 months?

13. Have you undergone clinical or laboratory tests because of breathlessness or wheezing or "whistling" in the chest or difficulty in breathing or asthma attacks in the past year?

14. How many times have you undergone these clinical or laboratory tests?

Spirometry ….

Skin allergy tests............

Blood testing for allergic.....

Chest radiograph or sinus ….

General blood analysis ….

CT lung …..

Methacholine challenge test …..

15. Are you currently employed?

16. How many days have you skipped work because of breathlessness or wheezing or "whistling" in the chest or difficulty in breathing or asthma attacks in the past year?

17. Are you currently taking any medicines including inhalers, aerosols or tablets for asthma?

17.1 Short or long-acting beta2 agonists

17.2 Which one?

17.3 How often have you used it over the last 3 months?

never

some of the days

most of the days

Whenever needed

17.4 How many times have you used it in the past 30 days?

18. Inhaled anticholinergics

18.1 Which one?

18.2 How often have you used it over the last 3 months?

never

some of the days

most of the days

Whenever needed

18.3 How many times have you used it in the past 30 days?

19. Inhaled corticosteroids (including combinations with beta 2 agonists)

19.1 Which one?

19.2 How often have you used it over the last 3 months?

never

some of the days

most of the days

Whenever needed

19.3 How many times have you used it in the past 30 days?

20. Oral methylxanthines

20.1 Which one?

20.2 How many times have you used it in the past 30 days?

21. Oral corticosteroids

21.1 Which one?

21.2 How many times have you used it in the past 30 days?

22. Oral Antileukotrienes

22.1 Which one?

22.2 How many times have you used it in the past 30 days?

23. Have you ever had a problem with sneezing, a runny or a blocked nose when you did not have a cold or the flu? NO YES

24. Have you used any nasal sprays for the treatment of your nasal disorder?

25. Have you ever had eczema or any kind of skin allergy?

26. Do you keep a cat?

26.1 Is your cat (are your cats) allowed inside the house?

26.2 Is your cat (are your cats) allowed in the bedroom?

27. Do you keep a dog?

27.1 Is your dog (are your dogs) allowed inside the house?

27.2 Is your dog (are your dogs) allowed in your bedroom?

28. Do you keep any birds?

28.1 Are any of these birds kept inside the house?

29. Gender

Man

Woman

30. Age

31. Education

32. Weight (Kg)

33. Height (cm)

|  | FEV1 (L) FEV(% pred.) | | FVC (L) FVC(% pred.) | | PEFR (L/s) | FEV1/ FVC (%) |
| --- | --- | --- | --- | --- | --- | --- |
| Before |  |  |  |  |  |  |
| After |  |  |  |  |  |  |
| Reversibility |  | | | | | |
